# Micro-computed tomographic evaluation of endodontic ledge position in relation to canal curvatures

**DOI:** 10.1186/s12903-022-02531-5

**Published:** 2022-11-10

**Authors:** Elio Berutti, Mario Alovisi, Edoardo Moccia, Massimo Carossa, Giovanni De Caro, Andrea Roccuzzo, Damiano Pasqualini, Nicola Scotti

**Affiliations:** 1 Department of Surgical Sciences, Dental School, Endodontics, University of Turin, via Nizza, 230, 0126 Turin, Italy; 2 Department of Periodontology, School of Dental Medicine, University of Bern, Bern, Switzerland; 3 Politecnico di Torino, Turin, Italy

**Keywords:** Micro-CT, Endodontic ledge, Mandibular molars, Canal curvature, Shaping aberrations

## Abstract

**Background:**

Endodontic ledge (EL) formation is the most common complication of endodontic treatment. Although various etiological factors have been identified, canal curvature is the most significant variable correlated with EL formation. The aim of this micro-computed tomographic (micro-CT) study was to evaluate EL position in the mesial canals of the lower molars in relation to the degree of canal curvature.

**Methods:**

Forty intact mandibular first molars with independent mesial canals with 20°–40° primary mesio-distal curvature, 10°–30° buccal-lingual canal curvature and 4 < r ≤ 8 mm main curvature radius were selected. Working length was measured with a K-File #10 and a high resolution pre-operative micro-CT analysis was performed. Ledges were created at the point of maximum canal curvature using stainless steel K-Files #30–35, alternating irrigation with 5% NaOCl and 10% EDTA. A post-operative high-resolution micro-CT analysis was then completed. Pre- and post-operative images were analyzed. The angle (α) formed between the vector passing through the geometric center of the EL and the center of the original canal lumen and the line joining the centers of the mesio-buccal and mesio-lingual canal orifices was calculated, and a descriptive statistical analysis was achieved. The α angle values were analyzed in relation to canal curvature using Kruskal-Wallis and post hoc Dunn’s tests. The level of significance was set at *P* < 0.05.

**Results:**

The α angles appeared inversely proportional to canal curvatures in the buccal-lingual and mesio-distal projections. The mean α angle was 36.4° (standard deviation 10.64; 95% confidence interval 34.1–40.9).

**Conclusion:**

Within the limitations of this study, endodontic ledges develop in the opposite direction to the three-dimensional canal curvature and their position is influenced by the degree of curvature. Clinically, the α angle values may be related to the recommended direction to manage endodontic ledges.

## Background

Successful endodontic treatment requires the determination and maintenance of a correct working length (WL), appropriate shaping, disinfection, and a three-dimensional (3D) filling of the root canal system [1, 2]. One of the most common complications occurring in clinical practice is the formation of endodontic ledges (EL). An EL is defined as a deviation from the original canal curvature that does not communicate with the periodontal ligament, and which has the potential to negatively influence outcomes [3].

Achievement of the correct WL may be compromised in the presence of an EL, resulting in a residual bacterial load in the apical root canal portion [3]. Consequently, formation of an EL can be directly linked to the development or persistence of an apical periodontitis, resulting in a negative outcome of endodontic treatment [3–5]. Previous studies reported that intraoperative complications such as incorrect working length is a significant outcome predictor, especially for teeth with a preoperative radiolucency [4, 5]. Clinicians should try to pass the ledge in such cases, with apical surgery the only alternative where this is not possible [6].

Kapalas et al. [7] observed that 51.5% of canals treated by students, and 33.2% of canals treated by endodontists may present with an EL. Although a variety of etiological factors have been identified [7–9], including coronal interferences [10] and inadequate WL determination and shaping, the root canal curvature is the most significant factor correlated with EL formation [3]. Indeed, Green and Krell [11] observed that the incidence of EL is significantly increased in canals with a curvature greater than 20°.

While previous studies have described the mean curvature range of the lower first molar canals [12, 13], EL position in relation to canal curvature has not been yet investigated but may help to clinically manage endodontic treatment in the presence of EL. The aim of this micro-computed tomographic (micro-CT) study was to evaluate EL position in the mesial canals of the lower molars in relation to the degree of canal curvature. The null hypothesis was an absence of correlation between the degree of curvature and EL position.

## Methods

Mandibular first molars with a fully formed apex that had not previously undergone endodontic treatment, and that had been freshly extracted for periodontal reasons, were selected in accordance with the local ethics committee. The patients provided an informed consent for their extracted teeth to be used for research purposes. The teeth were free of caries, cracks, and artificial alterations. A sample size of 40 was calculated with G*Power 3.1.4 (Kiel University, Kiel, Germany) ANOVA test to set the study power at 80% considering an alpha-error = 0.05.

After debridement of the root surface, the samples were immersed in a 0.01% NaOCl solution (Niclor 5, OGNA, Muggiò, Italy) at 4 °C for 24 h and then stored in saline solution. The teeth were placed on a customized support to perform a preliminary low resolution micro-CT scan to obtain an overall outline of the canal anatomy and to select teeth that met the inclusion criteria (SkyScan 1172, Bruker micro-CT, Kontich, Belgium). Preliminary scans were conducted as follows: 450 projections through a 225° rotation (180° plus cone angle of the X-ray source) using a 1.0 mm thick aluminum filter, voltage = 100 kV, current = 80 µA, source-to-object distance = 80 mm, source-to-detector distance = 220 mm, pixel binning = 8 × 8, exposure time/projection = 0.2 s. Axial sections were reconstructed with isotropic voxels and morphological parameters of the mesial canals were obtained. Mesio-buccal (MB) separated canals with a length of 12 ± 2 mm from the canal orifice to the apical foramen, 20°–40° primary mesio-distal curvature, 10°–30° buccal-lingual canal curvature and 4 < r ≤ 8 mm main curvature radius were selected [12–14]. The point of maximum curvature was located within the middle third of the root canal both from the proximal and buccal view. Teeth with confluent canals in the middle portion, accentuated isthmuses, significant calcifications, or double curvatures were excluded, as were any not concurring with the inclusion criteria described above. Of 51 teeth assessed for inclusion, 11 were excluded due to anatomical features and 40 were included in the micro-CT study.

A single expert operator with over ten years of experience in endodontics who was blinded to the objective of the study prepared the samples. Traditional pulp chamber opening was performed and, after checking the canal patency, the WL was measured in the MB canal with a K-File #10 and an operating microscope at 10X magnification (OPMI Pro Ergo, Carl Zeiss, Germany) until the tip of the instrument was visible at the canal apex. Ledges were then created at the point of maximum curvature within the middle third of the root canal through aggressive instrumentation of the MB canal with stainless steel K-Files #30–35, alternating irrigation with 5 ml 5% NaOCl and 10% EDTA administered with a 30-gauge needle syringe.

**Fig. 1:**
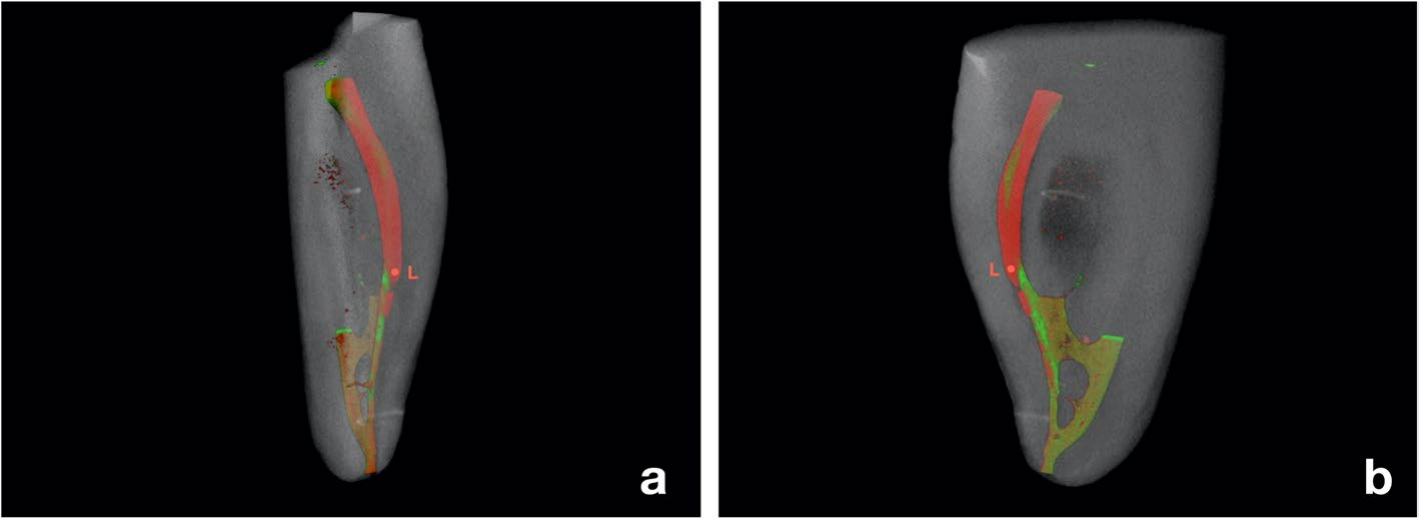
Micro-CT scans matching of pre- and post-operative volumes from a buccal-lingual (**a**) and mesio-distal (**b**) projection of the mesial canals of a lower molar with an endodontic ledge (L) in the mesio-buccal (MB) canal. The ledge is represented on the outside of the three-dimensional curvature of the instrumented MB root canal portion (red). The remaining not instrumented canal volume is highlighted in green

The samples were scanned at a high spatial resolution before and after ledge creation (SkyScan 1172, Bruker micro-CT, Kontich, Belgium) to assess positioning of the EL in relation to the original canal lumen and the degree of canal curvature (Fig. 1). The micro-CT scanning parameters before and after ledge creation were as follows: 2400 projections at 100 kV and 100 µA, with a focus point of 8 μm. The focus-object and object-detector distances were 98 and 218 mm, respectively. The total scan time was 4 h. High resolution 16-bit TIFF images were acquired. This resulted in a volume of approximately 1000 × 1000 × 1000 isotropic voxels containing a length of 9.2 μm.

Nrecon software (Bruker, Kontich, Belgium) was used to reconstruct the cross-sectional micro-CT images using the Feldkamp algorithm and standard parameters for *beam hardening* (60%) and *ring artifact correction* (7%). The native SkyScan data were automatically recognized to enable the superimposition of the pre- and post-operative volumes and cross sections trough profiles matching. The orthogonal cross sections were extrapolated using the DataViewer (Extron, Amersfoort, Nederlands) with the 3D recording function. Each instrumented root canal was dynamically studied by observing the high-resolution 3D rendering and the orthogonal transverse sections. The cross sections were analyzed in relation to the EL through a plane perpendicular to the major axis of the canal, and in relation to the canal orifice through a plane tangent to the pulp chamber floor.

**Fig. 2:**
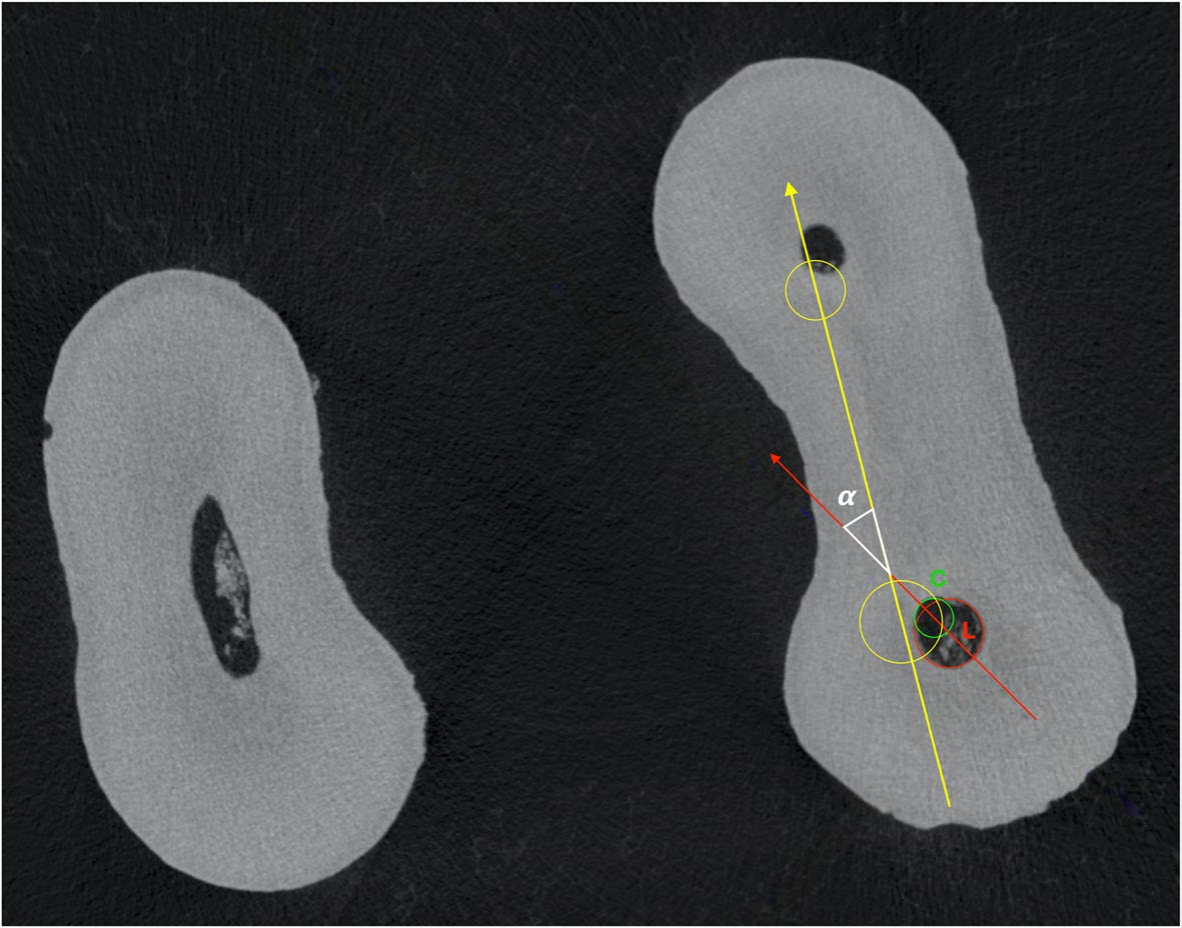
The vector drawn between the geometric center of the endodontic ledge (L) and the center of the original canal lumen (C) (red line). The internal angle (α) is described between this vector and the line joining the centers of the mesio-buccal and mesio-lingual root canal orifices (yellow line)

**Fig. 3:**
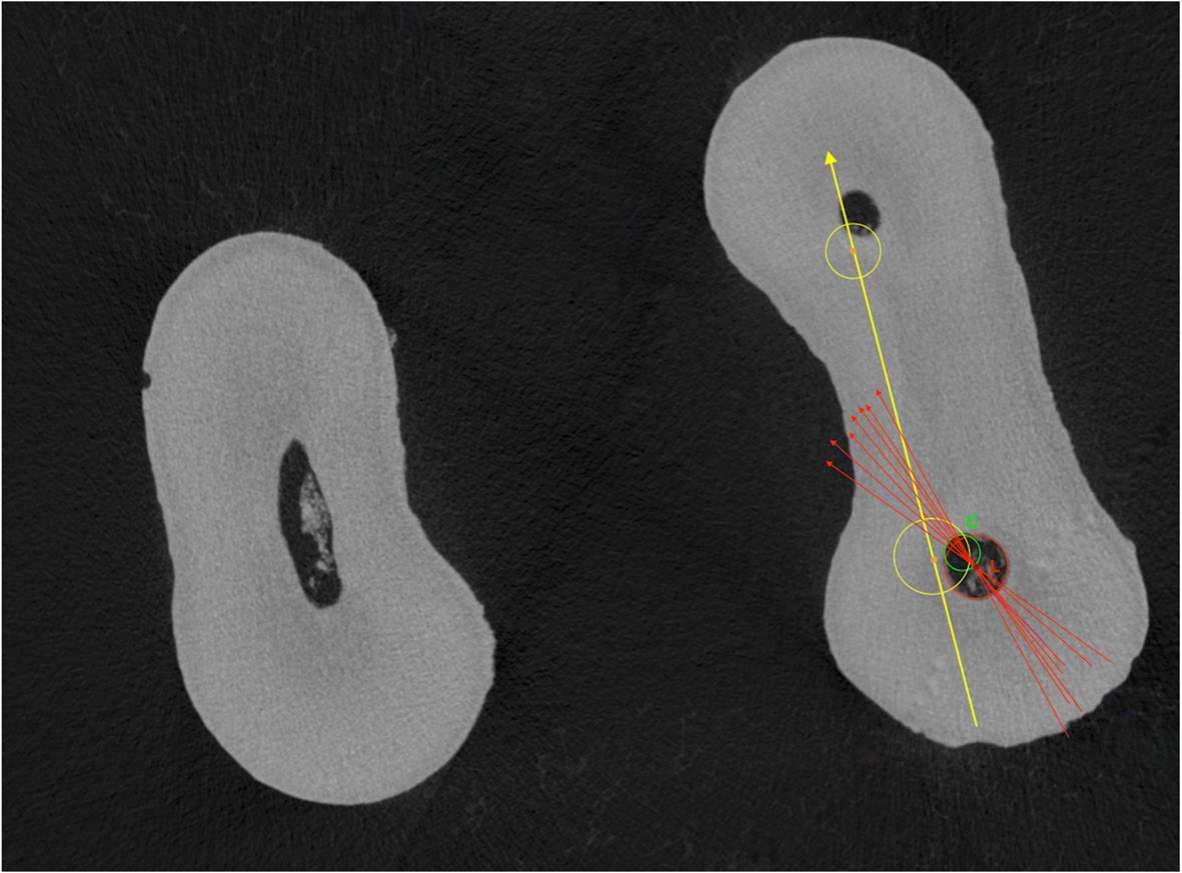
Vectors passing through the geometric center of the endodontic ledge (L) and the center of the original canal lumen (C) (red lines). The internal angle (α) is formed between these vectors and the line joining the centers of the mesio-buccal and mesio-lingual root canal orifices (yellow line) through a plane tangent to the pulp chamber floor. The red vectors represent a range of α angle values from different samples to highlight their common direction through the center of the pulp chamber floor

The cross sections were imported in JPEG format and analyzed with ImageJ 1.43u 64-bit software (National Institute of Health, Bethesda, USA). An automatic threshold algorithm (MT-minimum threshold algorithm) was used to eliminate the possibility of manual errors [15]. The geometric center of the ledge (L) was identified in the post-operative scans while the center of the original canal lumen (C) was visualized in the corresponding pre-operative sections. After the pre- and post-operative cross-sections superimposition, a vector passing through L and C was calculated for each sample (Fig. 2). Afterwards, the center of the mesio-buccal (MB) and mesio-lingual (ML) canal orifices was determined on the pre-operative scans in the sections corresponding to the pulp chamber floor. The internal angle (α), formed between the vector passing through L and C and the line joining the centers of the MB and ML canal orifices was measured once the geometric overlap between section was corrected to maintain the original spatial coordinates (Figs. 2 and 3).

A descriptive statistical analysis ​​was performed and the α angle values were analyzed in relation to canal curvature using Kruskal-Wallis and post hoc Dunn’s tests. The level of significance was set at *P* < 0.05.

## Results

The samples were grouped according to curvature of the MB canals in buccal projection (Table 1). Fifteen samples had a curvature in the range of 20° ≤ x < 27°, 13 in the range of 27° ≤ x < 34° and 12 in the range of 34° ≤ x < 40°. All the tested canals showed a greater mesio-distal than buccal-lingual root canal curvature.

**Table 1:** Mean α angle values in relation to lower molar mesio-buccal canal curvature in proximal and buccal projections (n = 40). The α angle values were significantly related to canal curvature in the buccal-lingual (*P* = 0.032) and mesio-distal (*P* = 0.041) projections. Different superscript letters (^a,b,c^) in the same column indicate significant differences between groups (*P* < 0.05)

Ranges canal curvatures in buccal projection (°)	Mean canal curvature in proximal projection (°)	Mean α angle (°)
20 ≤ x < 27	18.3^a^	44.6^a^
27 ≤ x < 34	19.4^b^	38.2^b^
34 ≤ x < 40	23.1^c^	36.5^c^

The α angle values were significantly related to canal curvature in the buccal-lingual (*P* = 0.032) and mesio-distal (*P* = 0.041) projections (Table 1). In particular, the α angle appeared inversely proportional to canal curvature for both mesio-distal and buccal-lingual projections. The mean α angle value was 36.4 degrees (standard deviation 10.64; 95% confidence interval 34.1–40.9).

## Discussion

This study suggests that the position of EL formation is related to the degree of canal curvature. The null hypothesis was rejected on the basis that a statistically significant correlation was described between EL position and the lower molar mesial canal curvature. These findings suggest a clinical method for the prediction of endodontic ledges’ position to facilitate the clinical management.

Carrying out a minimally invasive shaping while respecting the original canal anatomy in the absence of procedural aberrations, such as EL, is the main challenge for endodontics [16]. Modern mechanical instruments have evolved to improve shaping procedures and to simplify treatment for expert and non-expert operators [17–19]. A previous study [20] concluded that there is no correlation between an operator’s experience and their shaping ability with rotary instruments. However, the clinician’s skill still plays a major role in overcoming complications such as the occurrence of an EL [3, 21]. In the presence of an EL, a manual K-File should be manually bent to pass the ledge and scout the canal to locate the original canal patency. In general, root canal scouting is based on the operator’s manual sensibility, without information regarding search location in relation to EL position. This study aimed to describe EL positioning in relation to the canal curvature and to an easily detectable reference point during clinical practice, thereby facilitating the management of this type of aberration.

An extracted tooth model tends to be transferable to the clinical situation due to the similarity of experimental conditions [22]. Homogeneity of pre-operative characteristics of the selected samples is essential to ensure an adequate study standardization [18]. Mandibular molars tend to require the most involved endodontic treatment of any teeth, and their complex anatomy often leads to procedural aberrations [23, 24]. The mesial root of the lower molars is usually curved in both the mesio-distal and buccal-lingual observation planes [8, 25], where the canal may have an accentuated curvature not visible in periapical radiographs [26]. Gu et al. estimated the mean curvature of the mesio-buccal root to be 24.34° in the buccal-lingual projection and 16.60° in the mesio-distal projection, and for the curvature to be directed distally and towards the tooth center [12]. Villas-Boas et al. described high anatomical variability of the lower molar mesial roots, in terms of the presence of isthmuses, curvatures, and the canal Sect. [27]. As a result, lower molar mesial canals are primarily affected by canal aberrations during the scouting, glide path and shaping phases [3].

If the WL is not correctly maintained during instrumentation, residual pulp tissue and dentine debris may accumulate in the apical portion of the root, resulting in a canal block, even if a continuous irrigation with 5% NaOCl and 10% EDTA is ensured [28–31]. In this situation, a ledge may be created in the immediate coronal portion of the canal, especially if a rigid endodontic instrument is pushed against the canal walls to overcome the obstacle [3]. Rigid endodontic instruments, such stainless-steel K-Files, display a high stiffness and an active tip, and tend to maintain a straight position that does not follow the canal curvature, thereby increasing the risk of ledge formation [3].

Previous studies have demonstrated that micro-CT scanning represents an effective, non-invasive, and reproducible method of evaluating root canal preparations [32, 33]. Micro-CT scans enable the identification of morphological changes associated with different biomechanical preparations, including canal transportation and dentin removal [33, 34]. Moreover, overlapping of pre- and post-operative root canal volumes allows for a 3D qualitative analysis with high resolution volumes [2, 35]. Indeed, micro-CT analyses are so accurate they can be superimposed on histological sectioning, with the advantage of non-invasive image acquisition [36].

In this study, micro-CT analysis was implemented to obtain clinically visible reference points for analysis of the EL position in relation to canal curvature. Therefore, the axis passing through the center of the canal orifices of the two mesial canals was selected as a clinical reference point, as it may be standardized and is easy detectable. The superposition of the two-dimensional cross-sectional images enabled the creation of a linear vector for the analysis of EL position with respect to this axis. The data reported that the canal lumen was located towards the inside of the 3D root canal curvature with an angular range of 36.4° ± 10.64° with respect to the axis joining the canal orifices (Fig. 3). The results demonstrate that the degree of canal curvature in the buccal-lingual view is directly proportional to that observed in the mesio-distal view, and that both ​​are inversely proportional to α angle. Consequently, as the canal curvature increases in periapical radiographs, it may be increasingly possible to locate the original canal lumen through the mesial isthmus with a bent manual K-File. Therefore, the possibility to predict the ledge position basing on periapical radiographs could be clinically useful to select the right approach to bypass this aberration with bent K-Files.

## Conclusion

Within the limitations of this study, endodontic ledges appear to occur in the opposite direction to the 3D canal curvature. To pass a ledge, it may be necessary to orientate a bent stainless-steel K-File towards the inside of the canal curvature, according to a pre-determined angle (α angle) which is dependent on the root canal curvature.

## Data Availability

The datasets used and/or analyzed during the current study are available from the corresponding author on reasonable request.

## Abbreviations

3D: Three-dimensional
Micro-CT: micro-computed tomography
EL: Endodontic ledge
WL: Working length
ML: mesio-lingual
MB: mesio-buccal
